# Eliminating Disparities and Achieving Health Equity Using Implementation Science

**DOI:** 10.1093/geroni/igab046.190

**Published:** 2021-12-17

**Authors:** Beth Prusaczyk, Ana Baumann

**Affiliations:** 1 Washington University School of Medicine in St. Louis, Saint Louis, Missouri, United States; 2 Washington University in St. Louis, Saint Louis, Missouri, United States

## Abstract

Eliminating health disparities and achieving equity are central to aging services, programs, and research, as we work to ensure older adults are treated equitably compared to their younger counterparts. Additionally, aging services, programs, and research are not immune from the structural racism and other inequities that plague all facets of our lives, and we must work to eliminate disparities within them as well. This presentation will discuss how Implementation science can be used to advance both of these fronts. Implementation science frameworks can be used to ensure multiple levels of context are considered, which is critical when working against something as pervasive and structural as racism. Implementation science can also guide the adaptation of evidence-based interventions for different populations, including for older adults or for different racial or ethnic groups. Furthermore, there are important ways health equity research can improve implementation science that advance the shared goal of eliminating disparities.

